# Research progress on diagnostic techniques for different *Babesia* species in persistent infections

**DOI:** 10.3389/fcimb.2025.1575227

**Published:** 2025-05-16

**Authors:** Zelin Jia, Yuliang Zhang, Donghui Zhao, Haifeng Wang, Ming Yu, Zhilin Liu, Xin Zhang, Jiayu Cui, Xueli Wang

**Affiliations:** ^1^ College of Animal Science and Technology, Inner Mongolia Minzu University, Tongliao, China; ^2^ Tongliao City Animal Quarantine Technical Service Centre, Tongliao, China; ^3^ Tongliao Institute of Agriculture and Animal Husbandry Sciences, Tongliao, China

**Keywords:** *Babesia*, diagnostic techniques, serological detection, molecular biology, research advances

## Abstract

Babesiosis, a zoonotic parasitic disease caused by *Babesia* protozoa, poses significant infection risks across mammalian species. Clinical manifestations in vertebrate hosts range from spontaneous abortion to fatal outcomes, with immunocompromised individuals potentially transmitting the pathogen through blood products or transplanted organs, thereby amplifying epidemiological risks. Effective disease management carries substantial public health implications for livestock production, companion animal welfare, and food safety in endemic regions. In global endemic zones, conventional diagnostic approaches combine morphological identification of *Babesia* spp. with complementary serological assays. Contemporary molecular diagnostics, particularly nucleic acid amplification techniques, have emerged as valuable adjunctive tools. A critical challenge in veterinary practice involves persistent subclinical carriers among treated livestock populations, necessitating precise parasite speciation for effective transmission control. This review synthesizes recent advancements in babesiosis detection methodologies, with particular emphasis on their implementation in clinical microbiology laboratories. This article introduces the latest progress in Babesiosis detection technology and its application in clinical microbiology laboratories, to provide a theoretical and practical basis for the comprehensive prevention and control of Babesiosis.

## 1 Introduction

Babesiosis, caused by apicomplexan parasites of the genus *Babesia*, represents the second most prevalent blood-borne parasitic disease in vertebrates after trypanosomiasis (Penzhorn, 2006; Cook and Puri, 2024). Since Victor Babes’ seminal description of bovine babesiosis in Romania (1888), over 100 *Babesia* spp.have been taxonomically identified. The primary transmission vector remains ticks, with secondary routes including erythrocyte transfusion, transplacental transmission, and organ transplantation (Wudhikarn et al., 2011; Obeta et al., 2020; Handel et al., 2021; Kumar et al., 2021). Babesiosis is most prevalent in rodents, carnivores, and cattle, and it is considered relatively rare for the general public to be infected with babesiosis (Dvoraková and Dvorácková, 2007; Jerzak et al., 2023). Clinically significant species are categorized by erythrocytic trophozoite dimensions: Small-form *Babesia* (1.0-2.5 μm): Includes pathogenic species *Babesia gibsoni* (*B. gibsoni*) and *Babesia microti* (*B.microti*); Large-form *Babesia* (2.5-5.0 μm): Encompasses *Babesia bovis* (*B.bovis*), *Babesia caballi* (*B.caballi*), *Babesia canis*(*B. canis*) and *Babesia bigemina* (*B.bigemina*) (Panti-May and Rodríguez-Vivas, 2020). Epidemiologically critical zoonotic strains include *B. microti*, *B. duncani*, and *B. divergens*. Regional distribution patterns reveal: Pastoral areas: *B.bovis*, *B. bigemina*, and *B. caballi*; Companion animals: *B. canis* and *B.gibsoni*; Wild animals: *B. microti*. As obligate vertebrate pathogens, *Babesia* infections pose substantial challenges to both public health systems and veterinary management practices.

## 2 Epidemiologically significant babesiosis

**Figure 1:**
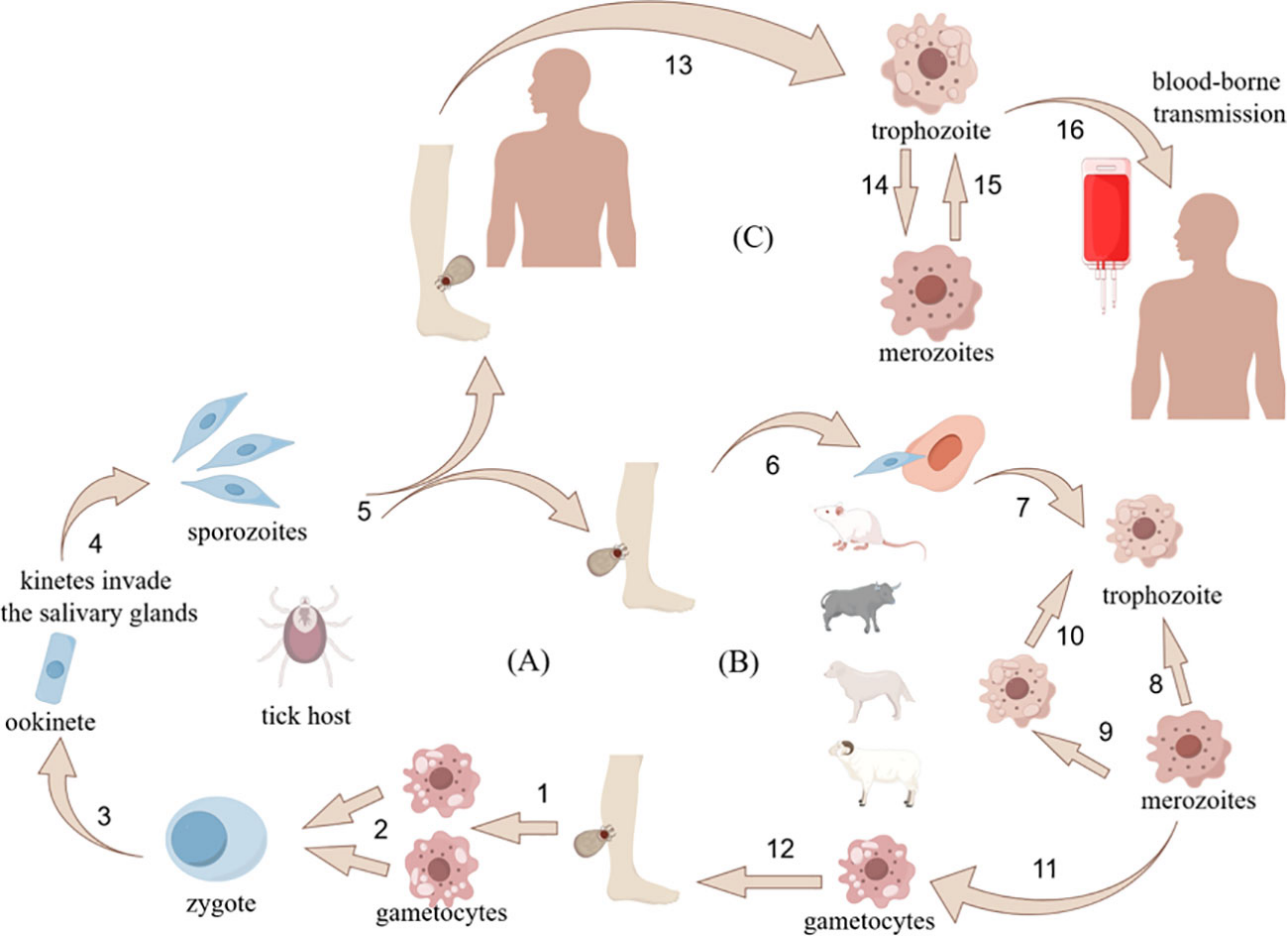
Detailed information on sexual and asexual reproduction of Babesia spp. Image created from the platform - Researchers' House license code exported ID:IWUAS50a2a.

Six *Babesia* spp. with confirmed zoonotic transmission include *B.microti*, *B. duncani*, *B. divergens*, *B. motasi* (KO-1 strain), *B. crassa*-like agent, and *B. venatorum*, alongside two genetically distinct pathogenic subtypes: *B. divergens*-like MO1 and *B. microti*-like protozoa (Kumar et al., 2021). Human babesiosis exhibits distinct geographic pathogen profiles: *B. microti* (asymptomatic to severe cases) and *B. duncani* (high-fatality infections) predominate in the Americas (Scott and Scott, 2018; Menis et al., 2021); *B. divergens* causes acute disease in splenectomized patients across Europe (Hunfeld et al., 2008); *B. crassa*-like and *B.venatorum* are hyperendemic in northeastern Asia (Jiang et al., 2015; Vannier and Krause, 2015; Jia et al., 2018; Chen et al., 2019); while Africa and Australia report indigenous strains (e.g., Egyptian variants) and imported pathogens (e.g., South African lineages) (Fang et al., 2015; Saleh et al., 2015; Kumar et al., 2021). *Babesia* spp. has a wide distribution and a complex life cycle. These apicomplexans maintain an obligate two-host lifecycle involving ixodid ticks (definitive hosts) and vertebrate reservoirs, with transmission dynamics diverging between morphological groups. Small *Babesia* spp. (*B.microti* clade) propagate through a rodent-tick-rodent cycle: larval ticks acquire parasites from infected rodents, transmit them during nymphal feeding, and reinfect new rodent hosts. Large *Babesia* spp. (*B.divergens* group) follow a ruminant-tick-ruminant cycle, where adult female ticks transmit parasites to grazing hosts, perpetuating through subsequent tick generations (Karbowiak et al., 2018). Sexual reproduction occurs exclusively within tick vectors, complemented by asexual replication in vertebrate hosts, as illustrated in **Figure 1**.

## 3 Clinical manifestations and pathogenic mechanisms

After the invasion of host erythrocytes by *Babesia*, multiple systemic pathological reactions are triggered by destroying the morphology of erythrocytes and inhibiting the oxygen-carrying function and hematopoietic ability. Clinical manifestations range from characteristic presentations—including hemolytic anemia, icterus, pyrexia, and respiratory distress—to potentially fatal complications such as splenic rupture and infarction. Notably, emerging evidence documents atypical immunopathological sequelae encompassing acute phase responses and secondary hemophagocytic lymphohistiocytosis (sHLH) (Milanović et al., 2020; Mojtahed et al., 2020; Santos et al., 2020; Jacobs et al., 2025). *Babesia* infection elicits host-specific pathophysiological responses across mammalian species. Rodent models demonstrate *B. microti*-induced hematological alterations characterized by accelerated coagulation kinetics and thrombotic predisposition, with gestational infections provoking placental vascular pathology through collagen deposition and erythrocyte-endothelial adhesion (Jasik et al., 2024). Bovine infections by *B. bovis* or *B. bigemina* clinically manifest as cerebral hyperemia, inappetence, lactation suppression, and hemoglobinuria crisis (Sivakumar et al., 2018). Canine cases present complex systemic involvement beyond febrile episodes and anorexia, progressing to renal impairment, pancreatic inflammation, and acute respiratory failure (“pulmonary shock”), with persistent subclinical carriage observed post-treatment (Muench et al., 2023; Antognoni et al., 2025). immunocompromised hosts, particularly splenectomized individuals, exhibit exacerbated disease trajectories confirming immune status-dependent pathogenesis (Mojtahed et al., 2020; Torianyk, 2021b; Kakoullis et al., 2025). Various species of *Babesia* have been reported to further exacerbate the risk of multi-organ dysfunction by activating the endogenous coagulation system and inducing immunopathologic responses (Welzl et al., 2001; Okła et al., 2014; Preena et al., 2021).

## 4 Detection of important babesiosis

In addition to the internationally recognized gold standard microscopic examination, detection techniques for babesiosis also rely on molecular diagnosis, genotyping, and serological methods. While molecular techniques, particularly nucleic acid amplification testing (NAAT), enable precise mapping of *Babesia* spp. distribution, population genetics, and co-infection epidemiology, their field utility remains constrained by instrumentation dependency and technical complexity. Conversely, seroepidemiological profiling captures immune response patterns across endemic regions, offering critical insights into antigenic variation mechanisms, albeit challenged by cross-reactivity with related apicomplexans, false-negative results during seroconversion windows, and prolonged antibody persistence (>12 months post-clearance). According to the diagnostic recommendations summarized in the analysis of the IDSA Guidelines for the Diagnosis and Management of Babesiosis, confirmation of a diagnosis of babesiosis requires a combination of peripheral blood smear microscopy (the gold standard) and PCR testing, and antibody testing alone is not recommended because antibodies may persist for more than 1 year after clearance of the infection (Krause et al., 2021).

### 4.1 Blood smear microscopy and emerging binding techniques

**Figure 2:**
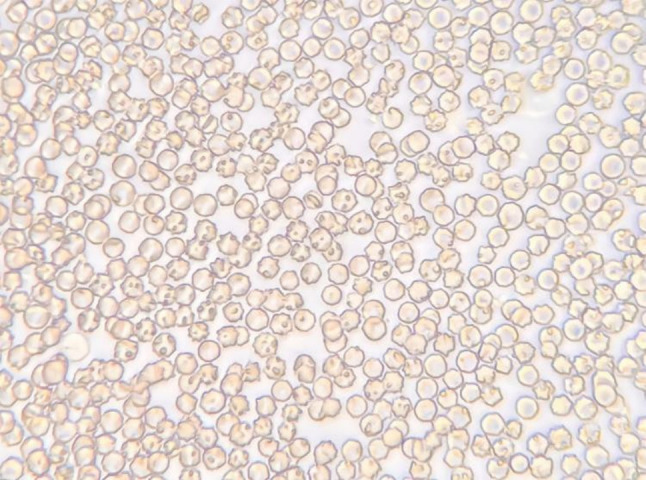
Microscopic examination of blood smears.

**Figure 3:**
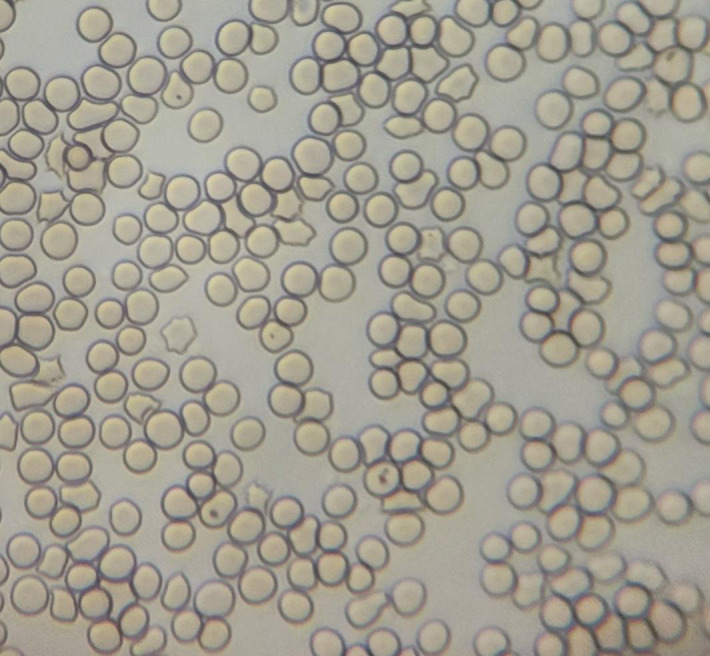
Microscopic examination of low-density Babesia parasitemia in blood smear.

A common method of detecting Babesia is to collect blood from terminal capillaries such as ear tips and tail tips to prepare blood smears, which are observed using a light microscope and the morphology of the parasite’s body (length of cleistothecia, etc.) is measured (**Figures 2**, **3**). In the acute infection phase, when the erythrocyte staining rate is high, only thin smears are required, conversely, thick smears prove essential during subclinical stages or post-acute phases when low parasitemia (<0.1%) challenges detection sensitivity (Alvarez et al., 2019; Dos Santos et al., 2021). While this method offers rapid field applicability, diagnostic accuracy suffers from morphological ambiguities between *Babesia* subspecies and *Plasmodium* spp., particularly in early infections lacking overt clinical manifestations. Notably, asymptomatic carriers often evade microscopic diagnosis despite harboring latent infections, with necropsy studies revealing striking tissue-specific parasitemia disparities—cerebral capillaries in *B. bovis*-infected hosts demonstrate 90-fold higher parasite burdens compared to peripheral blood (Everitt et al., 1986). One study successfully used cerebellar samples obtained from the foramen magnum of the occipital bone to achieve microscopic detection of persistently infected cattle (Hadani et al., 1982).

In recent years, recent advancements in diagnostic hematology integrate digital microscopy with artificial intelligence (AI) to revolutionize *Babesia* detection, exemplified by predictive models utilizing hematology analyzer parameters (ADVIA) for *Babesia canis* (*B. canis*) identification (Pijnacker et al., 2022). One study used a dual-model comparison strategy, i.e., constructing a conventional statistical model (CS) and a machine learning model (ML) based on data from infected cases diagnosed by blood smear or PCR, respectively. The results showed platelet count (PLT), mean platelet volume (MPV), and percentage of unstained cells (LUC%) as key predictors. The CS model achieved 84.6% sensitivity and 97.7% specificity (LR+ 36.78), while the ML model optimized via tree-based algorithms demonstrated perfect sensitivity (100%) with 95.7% specificity (LR+ 23.2). Algorithm-triggered alerts increase microscopic detection probability 37-fold versus routine screening, demonstrating scalable laboratory implementation. Complementing these developments, AI-augmented digital microscopy (IAAI) enables automated parasite speciation and parasitemia quantification, significantly reducing false-negative rates through computational erythrocyte analysis. This synergistic diagnostic ecosystem combines ML-driven prescreening with IAAI confirmation, leveraging routine hematological data for early infection alerts—particularly valuable during low parasitemia phases or microscopy oversights. While ML models enhance sensitivity through nonlinear multiparameter analysis, CS frameworks provide high-specificity decision support, collectively optimizing diagnostic workflows from initial suspicion to pathogen characterization (Durant et al., 2021).

### 4.2 Experimental inoculation and parasite culture

Experimental inoculation and heterologous diagnostic approaches have historically served as fundamental methodologies in *Babesia* research, tracing back to Victor Babes’ seminal application of Koch’s postulates through disease recapitulation in inoculated rabbits. Contemporary models employ species-specific vertebrate systems to mirror natural infection dynamics: *B. microti* pathogenesis is frequently studied in vole (*Microtus*) reservoirs, while *B. bovis* and *B. bigemina* isolation protocols typically utilize splenectomized bovine hosts via infected blood transfusion. The *B. divergens*-gerbil (*Meriones*) model has proven particularly valuable for investigating human-relevant pathogenesis. These controlled inoculation systems not only fulfill classical etiological criteria but also enable precise dissection of parasite-host interactions across zoonotic spectra (Krause et al., 1996; Gorenflot et al., 1998; Zintl et al., 2003; Holman et al., 2005). Experimental inoculation is an alternative diagnostic option for cases of hypoparasitemia, which occupied an important place in clinical practice until the advent of molecular biology tests (Krause et al., 1994). The Mongolian gerbil (*Meriones unguiculatus*) has emerged as a pivotal experimental model for *Babesia divergens* research, exhibiting acute and frequently lethal symptomatology distinct from bovine-hosted infections (Dkhil et al., 2014). Torianyk, Inna I et al. (Torianyk, 2021a) recent methodological innovations employ dual-host systems—combining Syrian hamsters (*Mesocricetus auratus*), gerbils, and murine models—to establish *in vivo* cultivation platforms through immune modulation. Host susceptibility stratification enables targeted propagation: hamsters maintain *B. microti* reservoirs while gerbils and mice sustain *B. divergens* cultures. Immunocompromised animal models coupled with multimodal diagnostics (clinical metrics: weight loss, motor dysfunction; laboratory confirmation: fluorescence microscopy/PCR) enhance *Babesia* detection sensitivity through standardized protocols, achieving parasitological confirmation at submicroscopic thresholds. This integrated approach not only facilitates pathogen isolation but also elucidates complication mechanisms, particularly erythrocyte adhesion-mediated vasculopathies and cytokine dysregulation. Transfecting Babesiosis into animals helps understand the immune mechanisms that clear or worsen the infection and the pathological processes leading to complications. While constrained by prolonged incubation periods (>7 days) and molecular confirmation requirements, these models provide unparalleled resolution for studying hypoparasitemic states and immune evasion strategies, establishing a critical methodological framework for both basic parasitology and translational therapeutic development.

The SCID murine model exhibits unique diagnostic utility in *Babesia* research through engineered erythrocyte replacement protocols. Immunocompromised mice receiving bovine red blood cell (BoRBC) transfusions alongside anti-murine erythrocyte monoclonal antibody treatment achieve rapid circulatory RBC substitution, creating a humanized hematological niche. Splenectomized SCID hosts inoculated with grazing calf-derived *Babesia* spp. develop marked parasitemia accompanied by pathognomonic manifestations including hemoglobinuria, hemolytic crisis, and neurological sequelae, enabling reliable pathogen isolation (Tsuji et al., 1995).

### 4.3 Immunological testing

Immunodiagnostic approaches constitute a critical pillar in comprehensive babesiosis management, enabling precise infection staging through serological antibody profiling, pathogen antigen detection, and immune complex characterization. Serological surveillance proves most effective during convalescent phases with established antibody responses, whereas antigenic assays are indispensable for early infection diagnosis before seroconversion or chronic carrier state identification. These immunological tools synergistically enhance blood product safety through donor screening protocols, significantly mitigating transfusion-mediated transmission risks. In clinical practice, integrated diagnostic algorithms combining antigen testing with PCR amplification demonstrate particular utility for confirming subclinical infections and hypoparasitemic cases, with subsequent microscopic verification ensuring diagnostic rigor. This multimodal strategy effectively bridges the diagnostic window period while addressing the inherent limitations of individual methodologies, thereby optimizing both epidemiological surveillance accuracy and therapeutic intervention timelines.

Pathogen-specific antigens serve as critical diagnostic biomarkers across infectious diseases, yet systematic antigen detection frameworks remain underdeveloped for babesiosis despite identification of multiple candidate targets. Take *B. microti* for example: Pioneering work by Lodes et al. first characterized *B. microti*’s immunodominant antigens through serological screening, establishing foundational targets for assay development (Lodes et al., 2000); Subsequent investigations by Homer’s team comprehensively validated the immunogenicity of novel antigenic candidates, advancing diagnostic reagent prototypes (Homer et al., 2003); Breakthrough research from Cornillot’s group identified the secreted antigen BmBAHCS1, demonstrating early IgM seroconversion at 4 days post-infection followed by IgG responses by day 8—a temporal profile enabling acute-phase detection (Cornillot et al., 2016). These cumulative discoveries highlight the evolving antigenic landscape while underscoring the need for standardized multi-antigen panels to address diagnostic window limitations and interspecies variability in *Babesia* infections.

Recent advancements integrating genomic screening with transcriptomic profiling have revolutionized *Babesia* antigen discovery, enabling systematic identification of immunodominant targets spanning secreted and membrane-anchored proteins. While serological antibodies remain pivotal biomarkers for exposure history, their diagnostic utility is constrained during early infection windows prior to seroconversion, complicating differentiation between active and resolved infections. In the early 1970s, experiments were conducted to establish indirect immunofluorescence assays (IFA) for antibody detection in animal models (Cox and Turner, 1970; Goldman et al., 1972; Leeflang and Perié, 1972), although antibodies can be effectively detected, there are limitations such as lack of early sensitivity, today only indirect fluorescent antibody assays (IFAs) are commercially available to detect antibodies against *Babesia divergens* in humans (Jian et al., 2010; Cheng et al., 2018; Wang et al., 2020a; Tijani et al., 2024). To contemporary enzyme-linked immunosorbent assay (ELISA) platforms. Early ELISA iterations utilized crude *Babesia* spp.-infected host lysates, progressively transitioning to recombinant antigen systems (Meeusen et al., 1985; Houghton et al., 2002; Loa et al., 2004; Luo et al., 2011). Recently, breakthrough innovations now achieve 100% diagnostic sensitivity through multiplex antigen panels combining BmMCFRP1, BmSERA1, BmPiβS, and BmBAHCS1 (Verma et al., 2020). Antigen-capture ELISA (e.g., Babesia duncani antigen capture assays (BdACAs)) further enhances specificity by targeting secretory antigens (BdV234, BdV38), achieving detection thresholds of 115 infected erythrocytes/μL while circumventing PCR false positives from residual DNA (Chand et al., 2024). These integrated approaches bridge critical diagnostic gaps, enabling precise infection staging from acute parasitemia to chronic carrier states.

Serodiagnostic innovations continue to evolve species-specific strategies for *Babesia* detection across host systems. In bovine babesiosis, a recombinant chimeric antigen (rMABbo) ELISA integrating *B. bovis* MSA-2c, RAP-1, and HSP20 epitopes achieves 95.9% sensitivity and 94.3% specificity, outperforming conventional single-antigen assays (Jaramillo Ortiz et al., 2018). Equine diagnostics benefit from multiplex antigen cocktails combining *B. caballi* rBC134f and rBC48t, advancing detection windows to 4 days post-infection through stage-specific epitope targeting (El-Sayed et al., 2023). Human diagnostics leverage peptide array technology to screen *B. microti* MSP2 and SA-1 antigenic motifs, demonstrating conserved immunoreactivity that minimizes false negatives from genetic variability, even in *Anaplasma phagocytophilum* co-infection scenarios (Tagliafierro et al., 2022). Despite these advances, serological limitations persist: IFA’s subjective interpretation and unstandardized reagents yield elevated false positives for *B. divergens* (Pokhil et al., 2020); while RAP-1 conservation between *B. bovis* and *B. bigemina* induces cross-reactivity (Jaramillo Ortiz et al., 2018). Notably, *B. microti* IFA exhibits superior specificity, with minimal cross-reactivity to other *Babesia* spp. except at low antibody titers (Chisholm et al., 1978). These findings underscore the necessity for standardized antigen panels and automated interpretation systems to harmonize diagnostic accuracy across zoonotic spectra.

Immunodiagnostic approaches for babesiosis demonstrate variable utility across infection stages and host systems. Complement fixation tests (CFT) targeting *Babesia bovis*-specific IgM antibodies show diagnostic value in acute infections but fail to detect chronic carriers due to transient complement-binding immunoglobulin responses (Mahoney, 1967; Ogunremi et al., 2007, 2008); Comparatively, indirect fluorescent antibody tests (IFAT) exhibit superior practicality through earlier seroconversion detection (3–5 days post-infection), reduced operational complexity (complement-independent), and lower costs (≈33% of CFT expenses), while maintaining >92% accuracy for *B. bovis* and *B. bigemina* identification (Kumar et al., 2003); Emerging rapid diagnostics like immunochromatographic tests (ICT) and automated fluorescence immunoassays (AFIA) enable high-throughput screening, though require geographic validation across diverse epidemiologic contexts (Moritz et al., 2017; Stuart Tayebwa et al., 2020; Andrade et al., 2024; Jongejan et al., 2024); The monoclonal antibody capture assay (mGPAC) offers precise diagnostic capabilities by targeting parasite-secreted antigens such as BmGPI12, specifically distinguishing active *Babesia microti* infections from prior exposures. This method demonstrates absolute specificity for *B. microti* without cross-reactivity to related species like *B. divergens* or *B. duncani*, making it particularly valuable for immunocompromised and elderly patient populations (Gagnon et al., 2022), With a detection limit of 7.3 pg/µl - 20 pg/µl, mGPAC surpasses both real-time PCR and microscopy in sensitivity and specificity for identifying active *B. microti* infections (Thekkiniath et al., 2018; Wilhelmsson et al., 2020; Montero et al., 2023), Its unique ability to differentiate current parasitemia from historical immune responses addresses critical diagnostic challenges in vulnerable cohorts, while maintaining rigorous species discrimination crucial for accurate clinical management; While traditional methods such as rapid conglutination tests (RCT) show strong concordance with ELISA/IFAT (κ=0.89) and favorable performance metrics (90.9% sensitivity, 97.6% specificity), their exclusion from World Organisation for Animal Health (OIE)-certified protocols stems from unstandardized procedures and inadequate quality control frameworks (Kappmeyer et al., 1999). Despite technological advancements, most serologic assays remain excluded from international diagnostic guidelines due to insufficient standardization. IFA persists as the serological gold standard, particularly valuable for transfusion-transmission investigations (Herwaldt et al., 2011; Moritz et al., 2016). Crucially, immunodiagnostics should complement—not replace—direct detection methods (microscopy/PCR), given their limitations in early infection windows (<5 days post-exposure) and immunocompromised hosts, coupled with prolonged antibody persistence post-cure (Vannier and Krause, 2012; Sanchez et al., 2016). This diagnostic synergy ensures accurate infection confirmation while addressing the temporal and immunological constraints inherent to antibody-based detection.

### 4.4 Molecular biology testing

Recent advances in molecular biology and immunological methods have enabled the development of nucleic acid probe assays for *Babesia* spp. identification. These assays employ primers and probes targeting conserved variable regions of parasite genes, allowing qualitative detection of parasite DNA in host blood samples. Complementing this approach, fluorescence *in situ* hybridization (FISH) technology specifically targets the 18S ribosomal RNA (rRNA) of *Babesia* spp. using labeled DNA probes, with positive hybridization signals visualized as green fluorescence under microscopy (Shah et al., 2020). The streamlined FISH protocol involves blood sample preparation with smear fixation reagents, followed by a 30-minute hybridization at 37°C and fluorescence detection, completing the entire diagnostic process within two hours (Shah and Ramasamy, 2022). This cost-effective platform requires minimal equipment, as conventional light microscopes can be adapted for fluorescence detection by installing LED light sources instead of specialized fluorescence microscopes. Unlike DNA-based PCR methods, FISH demonstrates superior performance by distinguishing viable from non-viable parasites while avoiding interference from PCR inhibitors in blood samples. The current assay panel covers zoonotic species (*B. microti*, *B. duncani*, *B. divergens*) and veterinary pathogens (*B. bovis*, *B. bigemina* in cattle, *B. caballi* in horses), with a detection limit of 57–58 parasites/μL. Notably, the technology maintains specificity even during co-infections with *Plasmodium* species or Lyme disease spirochetes. When combined with indirect fluorescent antibody (IFA) testing, this rRNA-targeted FISH approach enhances screening efficiency in endemic regions and shows particular promise for resource-limited settings due to its minimal infrastructure requirements and elimination of nucleic acid amplification steps.

Reverse line hybridization (RLB) has emerged as a robust platform for detecting tick-borne *Babesia* spp., exemplified by integrated systems like the Tekenscanner that enable rapid confirmation of host infections through species-specific probes (Stoltsz et al., 2020; Byaruhanga et al., 2023; Hoxha et al., 2025). Parallel advancements include Tick - Borne Disease Capture Sequencing (TBDCapSeq), a high-throughput probe technology capable of simultaneous genomic detection for 11 tick-borne pathogens across 50 samples per run. This method demonstrates exceptional sensitivity (1–10 genome copies), outperforming ultra-high-throughput sequencing (UHTS) by 25 to >10,000-fold and surpassing qPCR detection limits, making it particularly effective for large-scale surveillance. While its dual barcoding system reduces per-sample costs, TBDCapSeq requires optimized protocols for low-parasitemia specimens (e.g., increased blood volumes or sequencing depth) and faces limitations in detecting novel pathogens due to probe dependency on known sequences. Cross-reactivity risks from conserved rRNA gene regions further necessitate complementary genetic analyses for species confirmation (Jain et al., 2021).

These technological advancements position FISH as a rapid, cost-effective clinical/veterinary tool for detecting viable parasites, while TBDCapSeq and RLB provide high-throughput surveillance solutions. Future development should focus on probe specificity enhancements and integrated diagnostic workflows combining multiple techniques to address current limitations in pathogen coverage and analytical specificity.

Polymerase chain reaction (PCR) has established itself as a cornerstone technology for detecting *Babesia* infections and cryptic carrier states, driven by its exceptional specificity and sensitivity. Continuous methodological refinements have expanded its utility across diverse strains, including *B. caballi*, *B. microti*, *B. bovis*, and *B. bigemina*. Early conventional PCR systems (1990s) targeted conserved regions such as the 18S rRNA gene’s V4 domain in *B. bovis*, achieving detection limits of 10–100 organisms/mL (Fahrimal et al., 1992; Xu et al., 2018). The advent of reverse transcription PCR (RT-PCR) in the 2000s enabled transcriptional-level analyses, including *B. bovis* MSA-2c antigenic gene expression and *B. bigemina* rap-1 family transcriptional networks, while boosting sensitivity tenfold to 10 organisms/mL for low-parasitemia detection (Suarez et al., 2003; Wilkowsky et al., 2003; Colasante et al., 2024). Modern advancements center on real-time quantitative PCR (qPCR), exemplified by multiplex assays targeting the *sbp4* gene for *B. caballi* genotyping (Type A/B/C) with 5 copies/μL sensitivity (Giglioti et al., 2018); and extraction-free protocols enabling direct *Anaplasma/Babesia/Ehrlichia* triplex screening from diluted blood (43 copies/mL detection limit, 98% agreement with microscopy) (Patiño et al., 2024), Optimized qPCR achieves remarkable sensitivity for *B. bigemina* (1.5 infected RBCs/μL), surpassing microscopy by 100-fold (Stoltsz et al., 2020). Optimized nested PCR (nPCR) (Nicolaiewsky et al., 2001; Tian et al., 2015; Faria et al., 2017; Kumar et al., 2022) while nested PCR (nPCR) enhances *B. bovis* apocytochrome *b* gene detection to 2 parasites/0.5 mL blood – a 1,000-fold improvement over blood smears (Bhat et al., 2017; Radzijevskaja et al., 2018; Hong et al., 2019; Swei et al., 2019; Cai et al., 2024). Despite these advances, nPCR carries risks of underdetection in low-load samples and cross-species reactivity. Emerging approaches, such as genotype-specific qPCR (100% detection probability, 95% CI) (Venter et al., 2024). and semi-nested PCR coupled with SacII digestion (sensitivity to 0.00000012% parasitemia for *B. orientalis*) (Liu et al., 2007). Iterative optimizations in multiplex design, probe engineering, and workflow portability have collectively elevated PCR’s diagnostic precision for hypoparasitemia, mixed infections, and strain differentiation, solidifying its role in modern parasitology.

### 4.5 Isothermal amplification technology

Loop-mediated isothermal amplification (LAMP) has emerged as a transformative nucleic acid amplification technique, enabling rapid, equipment-independent detection of *Babesia* spp. under constant temperature conditions. This method excels in identifying early-stage infections (e.g., feline hosts), chronic cases (canines), and low-level or persistent parasitemia in diverse hosts including sheep, goats, and yaks (Guan et al., 2008; He et al., 2009; Mandal et al., 2015). Demonstrating superior analytical performance, LAMP achieves 100-fold greater sensitivity than PCR-agarose gel electrophoresis (PCR-AGE), 10-fold improvement over nested PCR (nPCR), and enhanced reliability compared to modified semi-nested PCR in baseline controls (Yang et al., 2016; Arnuphapprasert et al., 2023). Its detection threshold reaches 50 fg per reaction for *B. motasi* (Hong et al., 2019); with additional applications in species differentiation, such as distinguishing bovine *Babesia* from *Theileria* parasites (Tian et al., 2015). Recent innovations have expanded LAMP’s utility through hybrid platforms: LAMP-lateral flow dipstick (LAMP-LFD) integrates chromatographic strips for visual readouts, achieving 100-fold higher positivity rates than conventional PCR while maintaining absolute specificity for *Babesia* DNA, Complementary modifications like LAMP-LED and LAMP-UV incorporate portable light sources for result visualization, demonstrating 10-fold greater sensitivity than PCR-AGE for bovine babesiosis detection with 100% specificity (Yang et al., 2016; Schmidt et al., 2017). These advancements position LAMP as a field-deployable alternative to PCR, offering equivalent rapidity and sensitivity while eliminating the need for sophisticated thermocycling equipment.

Isothermal amplification technologies including recombinase polymerase amplification (RPA), recombinase-aid amplification (RAA), and cross-priming amplification (CPA) have demonstrated significant potential for field-deployable diagnostics of *Babesia* spp (Lei et al., 2020). RPA leverages recombinase-mediated strand invasion to amplify target sequences at low temperatures (37–42°C), achieving rapid detection thresholds of 22.5 copies/μL within 30 minutes when paired with lateral flow strips. Innovations like the *rpaBab264*-primer-based RPA-LFD system enable *B. vogeli* detection at 40°C in 10 minutes with comparable accuracy to conventional PCR. For *B. orientalis*, RPA targeting the mitochondrial *COXI* gene achieves 0.25 parasites/μL sensitivity in 15 minutes at 37°C—40-fold more sensitive than standard PCR—while maintaining specificity against apicomplexan relatives and host DNA (An et al., 2021; Nie et al., 2021; Onchan et al., 2022). RAA shares mechanistic similarities with RPA but operates at reduced costs, detecting 10 copies/reaction in 20 minutes (Lin et al., 2022). CPA distinguishes itself through cross-priming multiplex amplification, exemplified by a *B. duncani*-specific assay achieving 0.98 pg/μL sensitivity (~20 parasites/μL) at 59°C within 60 minutes via vertical flow strip visualization (CPA-VF), with 98.7% accuracy and a per-test cost of $0.20. Further optimizations yield CPA-VF sensitivities of 50 fg/reaction for *B. motasi* (5-fold superior to qPCR) and 320 fg/reaction for *B. bovis*, demonstrating field performance equivalent to PCR without cross-reactivity to co-circulating bovine pathogens (Wang et al., 2020b; Nian et al., 2024).

These platforms collectively address critical needs for rapid, low-resource *Babesia* detection, balancing high sensitivity, cost-effectiveness, and operational simplicity. TBDCapSeq demonstrates unparalleled analytical sensitivity (1–10 genome copies), surpassing qPCR, RPA, nested PCR (nPCR), FISH, and conventional PCR in detection limits. Isothermal amplification methods (LAMP, RPA, CPA) collectively outperform conventional PCR in both sensitivity and operational simplicity. Regarding processing speed, RPA achieves results within 10–30 minutes—the fastest among compared techniques—followed by FISH (2 hours), qPCR (~1 hour), and LAMP (~1 hour). Equipment needs vary substantially: FISH requires only a modified light microscope with LED illumination (lowest cost), while LAMP and RPA depend on basic thermostatic devices. qPCR necessitates fluorescence quantification instruments, and TBDCapSeq relies on high-throughput sequencers.

For clinical settings prioritizing rapidity and ease-of-use, FISH, LAMP, and RPA are optimal due to their minimal infrastructure demands and short turnaround times. Large-scale surveillance programs benefit from high-throughput platforms like TBDCapSeq, RLB, and multiplex qPCR. In scenarios requiring ultra-sensitive detection of low-parasitemia samples, nPCR and TBDCapSeq emerge as preferred choices, leveraging their enhanced limits of detection.

### 4.6 New technical applications for *Babesia* diagnostics

#### 4.6.1 High-throughput sequencing tool (*Haemabiome*)

The *Haemabiome* high-throughput sequencing platform represents a paradigm shift in *Babesia* diagnostics, employing dual-stage PCR amplification of conserved 16S/18S rDNA regions coupled with barcoding and Illumina MiSeq sequencing. This approach enables comprehensive pathogen identification through alignment with the SILVA ribosomal RNA database project (SILVA) reference database, achieving high-resolution detection of *Babesia* spp. alongside co-circulating genera such as *Theileria*. By integrating multi-pathogen detection capability with exceptional sensitivity, the system addresses the critical limitations of traditional single-target assays. Its capacity to resolve mixed infections and uncover genetic diversity within parasite populations positions *Haemabiome* as a powerful tool for epidemiological surveillance and complex clinical case analysis, particularly in regions endemic to multiple tick-borne pathogens (Yalcindag et al., 2024). Emerging omics technologies are revolutionizing *Babesia* diagnostics and pathogenesis research through multi-dimensional analytical approaches. Label-based high-throughput quantitative proteomics (HQP) employs tandem mass tagging (TMT) with *LC-MS/MS* to profile serum/urine proteomes, revealing early renal injury biomarkers like *NGAL* and *L-FABP* in canine babesiosis through integrated KEGG/GO pathway analysis (Bilić et al., 2023). Metagenomic next-generation sequencing (mNGS) provides hypothesis-free pathogen detection across diverse specimens (whole blood, plasma, urine), recently proving instrumental in diagnosing *Babesia*-induced hemolytic anemia from sputum samples—a case initially misattributed to Epstein-Barr virus complications (Lu et al., 2024). Metagenomic next-generation sequencing (mNGS) provides hypothesis-free pathogen detection across diverse specimens (whole blood, plasma, urine), recently proving instrumental in diagnosing *Babesia*-induced hemolytic anemia from sputum samples—a case initially misattributed to Epstein-Barr virus complications (Gyarmati et al., 2016; Schmidt et al., 2017; Vijayvargiya et al., 2019; Lu et al., 2024). At the cellular level, single-cell RNA sequencing (*scRNA-seq*) enables unprecedented resolution of *Babesia*’s asexual replication cycle through pseudotime gene expression mapping (Rezvani et al., 2022). Singh et al. (2023) complementing genomic advances exemplified by *B. duncani*’s fully sequenced genome with detailed epigenomic/transcriptomic annotations. Comparative multi-omics leveraging *Plasmodium* models reveal divergent drug sensitivity mechanisms between these apicomplexans, informing targeted therapeutic development (Si et al., 2023). Whole genome sequencing facilitates phylogenetic reconstruction and gene family evolution analysis, distinguishing *Babesia* lineages while identifying host-specific invasion determinants and virulence factors (Wang et al., 2022). Integrated genomic-proteomic workflows further enable rational vaccine antigen discovery through computational epitope prediction and functional validation (Liu et al., 2023).

#### 4.6.2 Microfluidics and biomedical engineering technology

Microfluidic platforms leveraging insulator-based dielectrophoresis (iDEP) offer a transformative approach for rapid *Babesia* detection by exploiting dielectric property differences between infected and healthy erythrocytes. Infection-induced structural alterations in red blood cell membranes—such as ridge formation—modify their polarization characteristics, enabling iDEP systems to separate parasitized cells via dielectrophoretic (DEP) forces in non-uniform electric fields. Validated through *GFP* labeling and Diff-Quik staining in *B. bovis* models, this technology processes 1 μL samples in <1 minute, concentrating 0.1% parasitemia specimens to 70% purity while achieving >98% healthy cell recovery at optimized voltages. With a sensitivity threshold of 0.1% parasitemia (5,000 parasites/μL), iDEP outperforms conventional microscopy but remains less sensitive than PCR (1–5 parasites/μL) (Adekanmbi et al., 2016). The platform employs a single-shell model to quantify infection-induced erythrocyte heterogeneity (e.g., membrane deformation, cellular swelling) through S-curve fitting, combining operational simplicity with analytical reliability. By eliminating time-consuming microscopy and complex nucleic acid amplification, iDEP significantly enhances transfusion screening efficiency and reduces babesiosis transmission risks. While currently validated for bovine *B. bovis*, adaptation to human pathogens like *B. microti* represents a critical next step for clinical translation (Oladokun et al., 2023).

#### 4.6.3 Biomarker tests

Emerging urinary biomarker assays and nanotechnology platforms are advancing early detection of *Babesia*-associated complications in veterinary medicine. The *uNGAL/uKIM-1* test quantifies urinary neutrophil gelatinase-associated lipocalin and kidney injury molecule-1 levels, demonstrating superior sensitivity for identifying acute kidney injury (AKI) in canine babesiosis compared to traditional renal markers. In dogs meeting International Renal Interest Society (IRIS) AKI criteria (serum creatinine elevation >0.3 mg/dL within 48 hours), *uNGAL* and *uKIM-1* levels showed significant elevation (*P*<0.001) even at IRIS grade I, proving particularly effective for detecting renal damage during non-azotemic stages (Asma Idress et al., 2024).

Complementing these biomarker approaches, nanoparticle-mass spectrometry (*NanoCage-MS*) employs engineered nanocages to capture *B. microti*-specific urinary proteins for subsequent mass spectrometric identification, enabling parasite detection through targeted proteomic signatures (Magni et al., 2020). While both technologies show promise for early clinical intervention—with *uNGAL/uKIM-1* enhancing AKI management and *NanoCage-MS* providing direct pathogen detection—their veterinary application requires further validation through large-scale, multi-etiology studies to confirm diagnostic accuracy across diverse infection scenarios.

#### 4.6.4 Genome and gene editing technologies

Next-generation sequencing platforms and CRISPR-based systems are driving transformative advances in *Babesia* genomics and diagnostics. Oxford Nanopore Technology (*ONT*) excels in resolving complex genomic architectures, while Illumina short-read sequencing provides high-fidelity assembly—complementary approaches that revealed *B. duncani*’s compact genome size and distinct phylogenetic lineage from *B. microti* and *Theileria* species (Wang et al., 2022). Targeted amplicon deep sequencing (*TADS*), though limited in parasite studies by host DNA interference and genetic diversity challenges, has been adapted through nested universal parasite detection (*nested UPDx*) to achieve qPCR-comparable sensitivity for discriminating *Babesia* from *Plasmodium* and *Trypanosoma* in blood samples (Flaherty et al., 2021).

CRISPR-Cas systems have emerged as precision tools for apicomplexan genome engineering, with Hakimi et al. pioneering *CRISPR-Cas9* applications in *B. bovis* for epitope tagging and gene replacement via Cas9/hDHFR plasmid systems (Hakimi et al., 2019). The compact *CRISPR-Cas12a* platform enables rapid field diagnostics through recombinase polymerase amplification (*RPA*) integration, detecting *Babesia* spp. via lateral flow strips in <2 hours with high specificity (Muriuki et al., 2024). Cas12 is a smaller nuclease that is easier to deliver to smaller parasites and has the potential for AAV-mediated delivery, providing an advantage for editing the apicomplexan genome (Webi et al., 2024). Due to the lack of RNAi inducible genes, RNAi has limitations in some apicomplexes, and Cas13, which targets RNA, offers new opportunities to study gene expression and regulation, but there is no Cas13 applied to *Babesia*.

These synergistic technologies—spanning long-read genomics, enhanced amplicon sequencing, and programmable nucleases—collectively accelerate functional genomics and field-deployable diagnostics for babesiosis management.

#### 4.6.5 Automated testing equipment and mass spectrometry

The *Sysmex XN-31m* automated hematology analyzer, originally developed for human malaria detection, has been successfully adapted for equine babesiosis diagnosis through fluorescence flow cytometry. This system identifies *Babesia*-infected red blood cells (iRBCs) by detecting parasite-specific fluorescent signatures, augmented by machine learning algorithms for pattern recognition. Capable of real-time parasitemia quantification, it reduces treatment decision times during acute infections while minimizing manual interpretation errors through automation (Ochi et al., 2024). With a limit of detection (LoD) surpassing conventional microscopy (>100 iRBCs/μL), the platform excels in high-throughput screening scenarios. Integrated multi-parameter analysis simultaneously evaluates routine hematological indices (red cell counts, hemoglobin levels), enhancing diagnostic comprehensiveness.

Complementing this approach, matrix-assisted laser desorption/ionization time-of-flight mass spectrometry (*MALDI-TOF MS*) enables direct pathogen identification from clinical specimens, recently extended to arthropod vectors (*Ixodes*, *Rhipicephalus*, *Amblyomma* ticks) and parasitic protozoa (Sánchez-Juanes et al., 2022). In canine babesiosis, the technology detects a unique 51–52 kDa serum protein biomarker absent in healthy dogs, demonstrating high specificity for *B. canis* diagnosis (Dzięgiel et al., 2016). While proven effective in malaria-endemic regions, broader *Babesia* applications require optimization of species-specific proteomic databases and standardized data protocols. Together, these automated systems bridge clinical and vector surveillance needs, offering scalable solutions for endemic disease management through rapid, operator-independent diagnostics.

#### 4.6.6 Flow Cytometry Combined with Artificial Intelligence

Emerging synergies between fluorescence technologies and artificial intelligence are advancing *Babesia* diagnostics through automated fluorescence flow cytometry (*FLC*), which enables direct parasite quantification from venous blood samples (Vanderboom et al., 2024). Current implementations require development of specialized machine learning (*ML*) algorithms to overcome the absence of dedicated analytical software, facilitating automated parasite recognition and quantification. The integration of fluorescence *in situ* hybridization (*FISH*) with flow cytometry presents a promising frontier, combining *FISH*’s species-specific detection capabilities with the high-throughput automation of flow systems to enhance both speed and analytical precision (Shah and Ramasamy, 2022). This hybrid approach could leverage optical enhancements to improve sensitivity while maintaining specificity, addressing critical gaps in rapid babesiosis screening. Future implementations may incorporate advanced imaging flow cytometry to simultaneously analyze morphological and molecular markers, enabling real-time parasite characterization. Such innovations position *FLC-AI* systems as transformative tools for clinical laboratories, offering scalable solutions that reduce operator dependency while improving diagnostic accuracy in both acute and surveillance settings.

#### 4.6.7 Other innovative technologies

Vibrational spectroscopy techniques are advancing *Babesia* diagnostics by capturing molecular fingerprints of host-parasite interactions across multiple scales. At single-cell resolution, *atomic force microscopy-infrared* sp*ectroscopy (AFM-IR)* enables nanoscale chemical imaging of infected erythrocytes, while *confocal Raman microscopy* maps molecular alterations during intracellular invasion. For population-level analysis, *attenuated total reflectance Fourier transform infrared* sp*ectroscopy (ATR-FTIR)* coupled with *partial least squares discriminant analysis (PLS-DA)* achieves 92.0% sensitivity and 91.7% specificity in <2 minutes post-sample processing. Rüther et al. optimized this approach for *B. bovis* through hemoglobin-depletion protocols and spectral signature modeling, enabling scalable detection from individual cells to clinical samples (Rüther et al., 2020).

Complementing these biochemical analyses, the *TFinder* deep learning system revolutionizes field diagnostics through automated *Babesia* quantification in bovine blood smears. This CNN-based platform, trained on 2,871 infected erythrocyte images, integrates multi-scale feature fusion and adaptive thresholding to achieve 98.0% sensitivity and 96.9% accuracy in qualitative diagnosis, with quantitative analysis reaching 99.7% specificity. By eliminating manual interpretation errors, *TFinder* detects parasitemia as low as 0.005%—20-fold below microscopy thresholds—while accelerating analysis 5–8-fold compared to conventional methods. Validated against 750 clinical samples, this first AI-driven solution for bovine tick fever meets medical device certification standards, with ongoing development focused on multi-pathogen detection models and cross-border field validation (Trindade et al., 2025). Together, these technologies exemplify the convergence of molecular phenotyping and artificial intelligence in transforming parasitic disease diagnostics.

## 5 Conclusion

We have compiled a list of important testing methods covered in the text (**Table 1** in the Attachments). Accurate species-specific diagnosis is fundamental for effectively preventing and controlling blood-borne parasitic infections. While microscopy remains the most accessible first-line tool due to low cost and rapid results, its clinical utility is constrained by limited sensitivity (particularly in low-parasitemia cases) and operator-dependent interpretation. Immunoassays address throughput limitations of conventional methods but require well-characterized species-specific antigens, with current platforms facing challenges of undefined antigen targets and cross-reactivity (**Table 2**). Serological approaches detect circulating antibodies yet suffer from persistent antibody retention post-recovery and false negatives in chronic carriers, compounded by reduced specificity when using crude antigen preparations. In contrast, nucleic acid amplification techniques (NAATs) offer superior sensitivity and speciation capacity, enabling discrimination between morphologically similar species like *Babesia* spp. and *Plasmodium* spp. Modern molecular advances now permit high-throughput screening and asymptomatic carrier identification – critical capabilities for endemic region surveillance. Current diagnostic integration strategies recommend: Primary screening: ELISA/ICT for clinical samples; Confirmatory testing: *In vivo* culture with microscopic examination/IFAT; Low-parasitemia cases: LAMP-assisted detection; Comprehensive analysis: PCR-based methods covering all parasitic life stages. *In vitro* culture systems serve dual purposes: pathogen isolation and supporting vaccine development through antigen characterization and drug susceptibility profiling. To minimize diagnostic errors, parallel testing with complementary assays is strongly advised. Future directions should focus on: Functional genomics of *Babesia* virulence factors through comparative interspecies transcriptomics; Multi-omics integration (proteomic/metabolomic) to elucidate gene expression networks governing life cycle progression; Next-generation assay development targeting differentially expressed biomarkers.

**Table 1:** Comparative analysis of important testing techniques.

Technical Name	Detection Principle	Detected Species	Advantages	Limitations	Detection Limit	Cross-Reactivity	Application Scenarios	References
Combinatorial Antigen ELISA	Detection of specific antibodies using BmMCFRP1/BmSERA1/BmPiβS/BmBAHCS1 antigen panel	*B. microti*	100% sensitivity	Relies on multi-antigen panel development	Signal/cutoff values: 0.245 (quadruple antigen) to 0.350 (triple antigen)	Not Applicable	High-sensitivity screening (human blood)	(Verma et al., 2020)
Antigen Capture ELISA (BdACAs)	Detection of circulating antigens targeting secreted BdV234/BdV38 antigens	* B. duncani *	Sensitivity 115 iRBCs/μl, avoids DNA residual false positives, signal proportional to parasitemia	Requires specific secreted antigens	Bd38ACA: 115 iRBCs/μl (10³ iRBCs/well); Bd234ACA: ~2×10³ iRBCs/well	No cross-reactivity with B. microti/B. divergens	Dynamic therapeutic evaluation	(Chand et al., 2024)
Recombinant Chimeric Antigen ELISA (rMABbo)	Detection of bovine Babesia antibodies using MSA-2c/RAP-1/HSP20 antigen integration	*B.bovis, B. bigemina*	95.9% sensitivity, 94.3% specificity	Potential cross-reactivity due to RAP-1 conservation	Cutoff ≥35% serum positivity (no direct pg/mL equivalent)	Cross-reactivity with B. bigemina (shared epitopes)	Bovine B. bovis screening	(Jaramillo Ortiz et al., 2018)
Cocktail Antigen ELISA (rBC134f+rBC48t)	Cocktail Antigen ELISA (rBC134f+rBC48t)	B. caballi *, T. equi*	Earliest detection at 4 days post-infection	Requires multi-stage antigen optimization	Full-dose: 0.38-0.71; Half-dose: 0.18-0.62 (OD values)	No cross-reactivity with T. equi	Early infection diagnosis	(El-Sayed et al., 2023)
Peptide Array Technology	Detection of human Babesia antibodies using conserved MSP2/SA-1 peptides	*B. microti *	Reduces genetic variation detection risks	Requires geographic strain validation	No traditional limit reported (OD/pg/mL)	Cross-reactivity not mentioned	Human Babesia serodiagnosis	(Tagliafierro et al., 2022)
Immunofluorescence Assay (IFA)	Detection of intracellular parasite antigens using fluorescently labeled antibodies	*B. divergens, B. microti*	Gold standard with low cross-reactivity (especially B. microti)	Subjective interpretation, poor standardization leading to false positives	IgG titer ≥1:128	Cross-reactivity not explicitly stated	Antibody screening/confirmation	(Pokhil et al., 2020)
Complement Fixation Test (CFT)	Detection of complement-binding IgM antibodies	*B.caballi*	High diagnostic value for acute infections	Cannot detect chronic infections, complex operation	Not Applicable	Cross-reactivity not mentioned	Supportive diagnosis of acute infections	(Ogunremi et al., 2008)
Indirect Immunofluorescent Antibody Test (IFAT)	Detection of specific antibodies using fluorescent secondary antibodies	*B. bigemina*	Earlier detection (3-5 days before CFT), lower cost (~1/3 of CFT)	Requires specialized fluorescence microscopy	Not Applicable	No cross-reactivity with other species	Bovine Babesia differentiation	(Andrade et al., 2024)
Immunochromatographic Test (ICT)	Rapid antigen detection using colloidal gold-labeled antibodies	*B.bigemina, B. bovis*	Simple operation, suitable for field screening	Need to validate host applicability range	Not Applicable	*No cross-reactivity between B. bigemina/B. bovis*	Large-scale epidemiological screening	(Stuart Tayebwa et al., 2020)
Monoclonal Antibody Capture Assay (mGPAC)	Detection of active *B. microti* infection targeting secreted BmGPI12 antigen	*B.microti*	100% specificity, distinguishes active vs. past infections	Limited human sample validation	7.3pg/μl-20 ng/mL	Not Applicable	Precision diagnosis in immunosuppressed patients	(Thekkiniath et al., 2018; Gagnon et al., 2022)
FISH Technology	Detection using fluorescent DNA probes targeting 18S rRNA genes	*B.microti*, *B.duncani*, *B.divergens*	Fast (within 2 hours), low cost, can distinguish between live and dead insects, no need for nucleic acid amplification, anti-PCR inhibitors	Requires fluorescence microscopy, species-specific probe design	57-58 parasites/μL	Cross-reactivity with *T. equi.*	Combined infection surveillance in resource-limited areas	(Shah et al., 2020)
Reverse Line Blotting (RLB)	Specific probe hybridization to target DNA	*B.bigemina*	High-throughput, multi-pathogen detection	Requires pre-designed probes, cannot detect novel pathogens	New probes 3× more sensitive than old ones (no pg/mL equivalent)	Not Applicable	Tick-borne disease monitoring, rapid host confirmation	(Stoltsz et al., 2020)
TBDCapSeq	High-throughput sequencing using dual-barcoding and probe capture technology	*B. microti*	Ultra-high sensitivity (1-10 genome copies), 50 samples per run, cost-effective	Cannot detect novel pathogens, requires optimization for low-load samples	1-10 genome copies	Cross-reactivity risk exists	Large-scale multi-pathogen screening	(Jain et al., 2021)
Conventional PCR	Amplification of conserved 18S rRNA gene regions	*B. bovis *	Basic species identification, standardized operation	Lower sensitivity (10-100 parasites/mL), requires electrophoresis	1-10 iRBCs/0.5 mL blood	No cross-reactivity with B. bigemina/A. marginale	Routine laboratory testing	(Fahrimal et al., 1992)
RT-PCR	Detection of gene expression (e.g., MSA-2c antigen gene) at transcriptional level	*B.microti*, *B.duncani*, *B.divergens*	10× higher sensitivity, gene expression analysis	Requires RNA stability, reverse transcription step	62.5 copies/mL	No cross-reactivity	Vaccine development, low-load infection analysis	(Colasante et al., 2024)
qPCR	Real-time fluorescent quantification (e.g., sbp4-based multiplex detection)	*B. bovis*, *B. bigemina*	Absolute quantification, genotyping (A/B/C types), high-throughput	High equipment cost, requires standard curve establishment	No specific sensitivity data provided	Not Applicable	Low parasitemia quantification, mixed infection typing	(Patiño et al., 2024)
Nested PCR (nPCR)	Two-round amplification for enhanced specificity	*B. microti *	Extreme sensitivity	Complex operation, contamination risk, cross-reactivity potential	3 fg/mL	Not Applicable	Screening for occult infections in very low load samples	(Cai et al., 2024)
LAMP Technology	Isothermal amplification (suitable for field use)	*Babesia* sp. BQ1 (Lintan), *Babesia* sp. Xinjiang-2005	Extreme sensitivity	Complex primer design, prone to non-specific amplification	0.02-0.2 pg genomic DNA	No cross-reactivity with Theileria/B. bovis	Low-cost alternative in primary labs	(Guan et al., 2008) (He et al., 2009)
RPA/RAA	Recombinase-mediated amplification (37–42°C)	RPA: *B. orientalis;* RAA: *B. microti*	Extremely rapid (10-30min), portable	Requires primer specificity optimization, slightly higher cost	RPA: 0.25 parasites/μL; RAA: 10 fg/μl	RPA: No cross-reactivity; RAA: No cross-reactivity with Toxoplasma/Plasmodium	On-site rapid screening (B. microti/B. orientalis)	(An et al., 2021) (Lin et al., 2022)
CPA	Cross-priming amplification with vertical flow strip detection	*B. motasi*	Ultra-sensitive, low cost (0.2 USD/test)	Longer reaction time (60min), requires temperature control	50 fg/reaction	No cross-reactivity with Theileria/B. divergens	Economic screening	(Wang et al., 2020a; Wang et al., 2020b)
Haemabiome	Two-round PCR targeting 16S/18S rDNA regions with barcode tags and MiSeq sequencing	B.bigemin,B. bovis	Simultaneous multi-genus detection (Theileria/Babesia), high sensitivity	Requires known pathogen database support	500 reads threshold for Theileria/Babesia genera	Not Applicable	Multi-genus pathogen detection	(Yalcindag et al., 2024)
High-Throughput Quantitative Proteomics (TMT-LC-MS/MS)	TMT-labeled serum/urine proteins analyzed by LC-MS/MS and bioinformatics	*B. canis*	Identification of NGAL/L-FABP as potential early biomarkers for renal injury	Requires mass spectrometry and bioinformatics expertise	No specific limit reported	Not Applicable	Biomarker discovery/validation	(Bilić et al., 2023)
Metagenomic Next-Generation Sequencing (mNGS)	Direct nucleic acid detection without predefined targets	*B. microti*	Simultaneous detection of bacteria/viruses/fungi/protozoa, novel pathogen discovery	Potential low sensitivity for rare pathogens, complex data analysis	SMRN ≥5 reads for positivity	Not Applicable	Complex infection diagnosis, novel pathogen detection	(Lu et al., 2024)
Single-Cell Transcriptomics (scRNA-seq)	Gene expression profiling of individual cells for pseudo-synchronous lifecycle analysis	*Babesia.duncani* (*WA1 strain*)	Reveals gene expression dynamics during asexual reproduction	High cost, complex operation	Single-cell resolution	Not Applicable	Parasite lifecycle research	(Singh et al., 2023)
Insulator Dielectrophoresis (iDEP) Microfluidics	Separation of infected/healthy RBCs based on dielectric properties using non-uniform electric fields	*B. bovis*	Rapid (<1min), low cost, μL-scale sample processing, 0.1% parasitemia sensitivity	Requires adaptation to human pathogens (e.g.,*B. microti*)	0.1% parasitemia (5000 iRBCs/μL)	Hypothetical human pathogen applicability not validated	Blood transfusion screening	(Adekanmbi et al., 2016)
Urine Biomarker Detection (uNGAL/uKIM-1)	Measurement of urinary NGAL/KIM-1 levels for acute kidney injury (AKI) detection	*Babesia canis,B. vogeli*	Early AKI identification	Requires standardized reference ranges	Serum creatinine ≥0.3 mg/dL within 48h	Not Applicable	Early renal injury diagnosis	(Asma Idress et al., 2024)
Nanocage-MS Technology	Nanocage-based capture of Babesia proteins in urine followed by mass spectrometry	*B. microti*	Specific detection of parasite protein biomarkers	Requires further clinical validation	1 pg/mL protein concentration	Not Applicable	Urine sample detection	(Magni et al., 2020)
Long-Read Sequencing (ONT) + Short-Read Sequencing	Hybrid assembly combining ONT for complex regions and Illumina for high accuracy	*B. duncani*	Resolves genome features (size/evolution), supports comparative genomics	High cost, complex data analysis	Single-copy gene variant detection	Not Applicable	Genome structural research, phylogenetics	(Wang et al., 2022)
Targeted Amplicon Deep Sequencing (TADS)	Nested UPDx reduces host DNA interference for improved parasite DNA detection	*B.microti, B.divergens, B.duncani, B.divergens-like variant MO1*	Detection limit comparable to qPCR, multi-species differentiation	Relies on conserved locus design	No specific limit reported	Not Applicable	Blood parasite detection	(Flaherty et al., 2021)
CRISPR-Cas	RNA-guided genome editing (Cas9/Cas12a)	*B.bigemina*	Precise gene editing, supports point mutations/gene replacement and rapid detection (combined with RPA)	Cas13 application not yet realized	10² DNA copies/μL	No cross-reactivity with B. bovis	Gene function research, rapid diagnosis	(Muriuki et al., 2024)
Sysmex XN-31m Automated Hematology Analyzer	Fluorescence flow cytometry with machine learning for infected RBC feature recognition	*B.caballi*	Real-time detection, better low-parasitemia sensitivity than microscopy	Requires species-specific blood sample adaptation (e.g., equine blood)	4.54 iRBCs/μL	No cross-reactivity with T. equi	Acute infection rapid diagnosis	(Ochi et al., 2024)
Matrix-Assisted Laser Desorption/Ionization Time-of-Flight Mass Spectrometry (MALDI-TOF MS)	Detection of specific serum protein fragments (e.g., 51-52 kDa)	*B.canis*	High specificity/sensitivity, supports tick species identification	Requires optimized Babesia proteome database	51-52 kDa protein marker	Not Applicable	Serum sample detection	(Dzięgiel et al., 2016)
Automated Fluorescence Flow Cytometry (FLC)	Direct detection of Babesia in venous blood using machine learning analysis	*B.microti*	Rapid quantitative detection	Requires dedicated software development	Not Applicable	Not Applicable	Blood sample detection	(Vanderboom et al., 2024)
Vibrational Spectroscopy (AFM-IR/Confocal Raman/ATR-FTIR)	Chemical fingerprinting of single cells/populations with PLS-DA analysis	*B. bovis*	Multi-scale detection (single cell to population), <2min analysis time	Requires hemoglobin removal pretreatment	ATR-FTIR: 92.0% sensitivity, 91.7% specificity	Not Applicable	Single-cell interaction analysis, rapid population screening	(Rüther et al., 2020)
TFinder AI System (Deep Learning)	Improved CNN architecture with multi-scale feature fusion and adaptive thresholding	*Babesia* spp.	Automated detection/quantification 突破 0.005% parasitemia limit	Requires multi-pathogen validation	98.0% qualitative sensitivity, 98.9% quantitative accuracy	Not Applicable	On-site rapid diagnosis of bovine babesiosis	(Trindade et al., 2025)

**Table 2:** The Protein Name and Accession Number of antigen genes.

Protein Name	Accession Number
BmMCFRP1	XP_012648612.1
BmSERA1	XP_012650223.1
BmPiβS1	XP_012649578.1
BmBAHCS1	XP_012648767.1
BdV234	BdWA1_002223-T1
BdV38	BdWA1_001730-T1
MSA - 2C	AY052542.1
RAP1	AF030062.1
HSP20	AF331455.1
rBC134f	BAC23026.1
rBC48t	BAA83725.1
